# 

**Published:** 1905-04-15

**Authors:** 


### ANNOUNCEMENTS.

LEWIS AND CLARK DENTAL CONGRESS.—This dental congress will be held in Portland, Oregon, July 17th to 20th, 1905, and promises to be the largest dental convention ever held west of the Rocky Mountains. Four or five adjoining States will omit their regular State meetings this year in order that they may join this congress. A low railroad rate of $56 from Chicago for the round trip, it is expected, will induce not a few eastern dentists to make the trip to the coast and join in the meeting. Committees in the interest of the convention have been appointed in every State and territory, and it is expected that these committees will do all they can to promote the congress. Dr. George L. Field, of Detroit, is chairman for Michigan. Dr. George E. Hunt, of Indianapolis, is chairman for Indiana. Dr. L. P. Bethel, of Columbus, is chairman for Ohio. Dr. G. V. Black, of Chicago, for Illinois, and Dr. W. E. Grant, of Louisville, for Kentucky.

MICHIGAN BOARD OF EXAMINERS.—This body will meet at Ann Arbor, May 16th, 1905. The first session will be called at 9 A. M., in the Dental College on the University campus. Address Dr. C. H. Oakman, Secretary, Cleland Building, Detroit.

—✦—

NATIONAL ASSOCIATION OF FACULTIES.—This body will meet at Buffalo, N. Y., Thursday, July 27th, 1905, at 2 P. M. The executive committee will meet at 10 A. M., the same day. The meeting will probably be held in the Iroquois Hotel.

—✦—

SOUTH Dakota Board of Examiners will meet at Mitchell, S. D., July 11th, at 1.30 P. M. Applications for registration with the fee of $10.00 must be in the hands of the secretary before July 7th, 1905. G. W. COLLINS, *Sec.*
Vermillion, S. D.

—✦—

WEST VIRGINIA BOARD OF EXAMINERS.—This board will meet June 7th to 9th, 1905, at Morgantown, W. Va. Candidates should file their applications by June 1st, with Dr. H. M. Van Vorhis, secretary, Morgantown, W. Va.

—✦—

KENTUCKY State Dental Association will meet May 15th and 16th, 1905, at Lexington, Ky. A cordial invitation is extended to all practitioners.

—✦—

EASTERN Indiana Dental Association will hold its thirty-fifth annual meeting at Greenfield, Ind., May 3rd and 4th, 1905. A splendid program of papers and clinics is being prepared. G. E. STEVENSON, *Sec.*

MISSOURI State Dental Association will meet in St. Louis, May 24th to 26th, 1905. An excellent program of papers and clinics will be presented, and all ethical members of the profession are cordially invited to attend.

SAM'L T. BASSETT, *Sec.*

—✦—

KANSAS STATE DENTAL ASSOCIATION.—The dates given for this meeting should be May 18th to 20th, 1905, and not May 17th to 19th, as printed in last month's REGISTER.

—✦—

NATIONAL ASSOCIATION OF EXAMINERS.—This body will meet at the Iroquois Hotel, in Buffalo, N. Y., Monday, July 24th, 1905, at 10 A. M., and continue in session until adjournment is ordered.

CHARLES A. MEEKER, *Sec.*
29 Fulton Street, Newark, N. J.

